# Comparability of Slack Water and Lagrangian Flow Respirometry Methods for Community Metabolic Measurements

**DOI:** 10.1371/journal.pone.0112161

**Published:** 2014-11-26

**Authors:** Emily C. Shaw, Stuart R. Phinn, Bronte Tilbrook, Andy Steven

**Affiliations:** 1 School of Geography, Planning and Environmental Management, The University of Queensland, Brisbane, QLD, Australia; 2 Oceans and Atmosphere Flagship, Commonwealth Scientific and Industrial Research Organisation (CSIRO), Hobart, TAS, Australia; 3 Oceans and Atmosphere Flagship, Commonwealth Scientific and Industrial Research Organisation (CSIRO), Brisbane, QLD, Australia; Centrum Wiskunde & Informatica (CWI) & Netherlands Institute for Systems Biology, Netherlands

## Abstract

Coral reef calcification is predicted to decline as a result of ocean acidification and other anthropogenic stressors. The majority of studies predicting declines based on *in situ* relationships between environmental parameters and net community calcification rate have been location-specific, preventing accurate predictions for coral reefs globally. In this study, net community calcification and production were measured on a coral reef flat at One Tree Island, Great Barrier Reef, using Lagrangian flow respirometry and slack water methods. Net community calcification, daytime net photosynthesis and nighttime respiration were higher under the flow respirometry method, likely due to increased water flow relative to the slack water method. The two methods also varied in the degrees to which they were influenced by potential measurement uncertainties. The difference in the results from these two commonly used methods implies that some of the location-specific differences in coral reef community metabolism may be due to differences in measurement methods.

## Introduction

The calcium carbonate (CaCO_3_) structure of coral reefs is formed by biogenic calcification among many different marine taxa, including scleractinian corals and coralline algae. For coral reefs to persist and grow, the rate of biogenic calcification must exceed the rates of physical destruction and chemical dissolution of CaCO_3_. Calcification occurs according to either of the following two reactions (dissolution is the reverse reaction), depending on whether seawater carbonate (CO_3_^2-^) or bicarbonate ions (HCO_3_^-^) are utilized [1] (Equations 1 and 2).
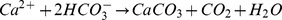
(1)




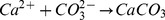
(2)Regardless of which equation is used, the total alkalinity (TA) of the seawater is reduced by 2 moles for each mole of CaCO_3_ produced through calcification, with the reverse being true for dissolution. This relationship between seawater alkalinity changes and calcification rate has been utilized for several decades in the alkalinity-anomaly technique to determine calcification rates for both organisms in aquaria and *in situ* for coral reef communities [2]–[5].

With changes in calcification rate of corals already observed [6], [7], and further declines predicted as a result of ocean acidification, climate change and local stressors [8], there have been several recent studies aimed at determining *in situ* calcification rates of coral reef communities [9]–[15]. The alkalinity-anomaly approach used in these studies allows the net calcification (gross calcification – dissolution of CaCO_3_) to be determined under natural conditions *in situ* at the scale of the reef community (i.e. incorporating calcification/dissolution in all organisms and sediment). The most common alkalinity-anomaly approaches are the slack water [16] and flow respirometry [17] methods, although variations using control volumes [9], [14], enclosures [18] and water residence time [19] have also been used (see [4] for a description of the methods).

Net community production (P_net_; i.e. photosynthesis – respiration) is also a key metabolic process on coral reefs and closely relates to net community calcification rates (G_net_), both in organisms and at the community scale [1]. P_net_ does not change seawater TA (apart from changes due to nutrient assimilation and release that are negligible in coral reef environments [2]), but does change the seawater dissolved inorganic carbon concentration (DIC). For every mole of organic carbon that is fixed through photosynthesis, seawater DIC is reduced by one mole (with the reverse being true for respiration). These known effects of P_net_ on seawater chemistry allow it to be calculated using the methods mentioned above for the alkalinity anomaly approaches.

Calcification rates from both the slack water and flow respirometry methods are calculated using the same general approach (Equation 3).
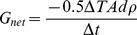
(3)where ΔTA is the change in salinity-normalized TA over the period of time between the two measurements (Δt), d is the water depth at the time of sampling and ρ is the density of the seawater. TA concentration is salinity-normalized to account for any changes in TA that occur from evaporation/precipitation so that the remaining ΔTA is assumed to be from calcification/dissolution.

Similarly, P_net_ is calculated as follows for both the slack water and flow respirometry methods (Equation 4).
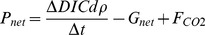
(4)where ΔDIC is the change in salinity-normalized DIC over the time period (Δt) and F_CO2_ is the air-sea flux of CO_2._

The difference between the slack water and flow respirometry methods is whether the seawater for which the measurements are being taken is flowing or not. The slack water method is a simplification of an Eulerian non-conservative transport model in which fluxes of DIC or TA are due to local changes, advection and dispersion [20], where the slack water method assumes no water flow (i.e. that the advection and dispersion terms are negligible). The flow respirometry method measures the upstream-downstream changes of the chemical properties in a parcel of water in a unidirectional current flow and can be performed using Eulerian or Lagrangian approaches [4]. Different physical characteristics of individual reefs mean that the flow respirometry and slack water methods are appropriate for different sites. The slack water method is appropriate for sites such as tidal pools or reef flats that become tidally isolated from surrounding flowing water at low tide. The flow respirometry method can be applied to reef areas that experience unidirectional tidal or wave-driven currents, such as reef flats and crests [4].

It is desirable to be able to compare community calcification rates of different reefs and the factors influencing calcification rates at different locations [11], [12]. Because the slack water method can generally only be used at low tide periods, diurnal curves of calcification rate are usually constructed from measuring calcification at several low tides over different times and days [16]. However, this approach does not allow for the full range of natural environmental conditions to be experienced while taking measurements only at low tide. For example, water flow conditions are not representative of other tidal stages and light/temperature conditions may be more extreme in the shallower low tide conditions. This could have important implications for the comparability of findings derived from the two methods, which are used under different flow conditions. Laboratory and *in situ* chamber studies have shown that rates of calcification and dissolution of coral reef sediments and calcification in reef organisms can be enhanced under higher water flow rates [21]–[23]. Therefore, it could be expected that the slack water method may result in measurements from times of relatively low metabolic rates compared with the flow respirometry method.

Few studies have compared different techniques for calculating *in situ* community metabolic rates on coral reefs. McGillis *et al*. [24] compared *in situ* net community production rates at Cayo Enrique Reef, Puerto Rico, using boundary layer and enclosure methods and found the two methods to give similar results. Gattuso, Frankignoulle and colleagues [25], [26] have compared air-sea CO_2_ flux measurements using both Eulerian and Lagrangian techniques at both Moorea and Yong Reefs. They found the Lagrangian method yielded flux estimates that were 1–2 orders of magnitude greater than the Eulerian method in both locations. The exact reason for the discrepancy was unknown, but the authors noted that the studies using the two different methods did not refer to the same communities or encompass the same reef area [26].

Despite the increasing number of *in situ* community calcification and production measurements occurring, and the popularity of the flow respirometry and slack water approaches, the comparability of these methods is largely unknown. The reef flat at One Tree Island (OTI), Great Barrier Reef (GBR), has tidal flow characteristics that allow periods of both slack water (during low tide) and unidirectional flow (during the outgoing tide) each tidal cycle. Here we exploit these characteristics to calculate metabolic rates using both the slack water and Lagrangian flow respirometry approaches, and compare the results from each of the two methods.

## Methods

Fieldwork was conducted under Great Barrier Reef Marine Park Authority permit number G11/34103.1. All measured and calculated data are available online [27].

### Study Site

**Figure 1:**
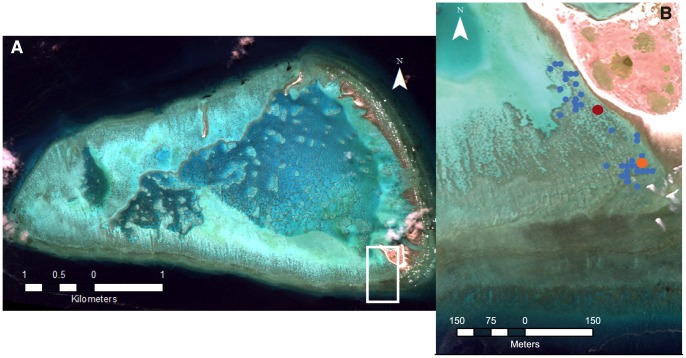
Study site. (A) One Tree Island and reef. (B) Reef flat study site showing sampling locations. The main slack water sampling site is shown in red and second slack water site shown in orange. Flow respirometry start and end points are shown in blue. Image data processed by the University of Wollongong. Worldview 2 data provided by Digital Globe.

OTI (23°30′S, 152°06′E) is a rubble cay in the Capricorn-Bunker group of the southern GBR. One Tree Reef is approximately 5.5 km by 3 km, with OTI on the windward side and three main lagoons within the reef area (Fig. 1a). The reef experiences semidiurnal tides and is isolated from offshore water at mid-low tide when the tide drops below the height of the reef crest [28].

The study occurred on the reef flat on the south-western side of OTI (Fig. 1). The study site was chosen as it has slack water periods every low tide and unidirectional current flow on the outgoing tide from the inner reef flat to the reef edge and thus could be used for both the slack water and flow respirometry community metabolic measurements. Live coral cover was estimated to be 25% based on two point intercept transects [29] that were performed in November 2013, across the length of the study area, perpendicular to the reef edge, with observations every 50 cm (total 600 data points).

### Sampling and analysis

Sampling occurred during 8–22 November, 2013. Slack water and Lagrangian flow respirometry methods, as described by Langdon et al. [4], were used to determine reef community metabolic rates. A main slack water site was designated, for which a total of 22 community metabolic rate measurements were performed at different times of a 24-hour day, where each metabolic rate measurement constitutes a pair of seawater samples. As the slack water method measures metabolic changes to seawater chemistry over a smaller spatial scale than the flow respirometry method, an additional slack water site further along the flow respirometry transect was also selected, where an additional five P_net_ and G_net_ measurements were taken (Fig. 1b). It was planned to take the same number of flow respirometry measurements as for the main slack water site, but the loss of two seawater samples during analysis and bad weather conditions on one sampling day resulted in 18 flow respirometry measurements. For the slack water method, seawater samples were collected at the sampling site at low tide and an hour each side of low tide. For the flow respirometry method, samples were collected around mid-tide on the outgoing tide. An initial water sample was collected and a Lagrangian drifter deployed to track the water flow along the transect. At the end of the transect a second water sample was collected and the amount of time elapsed between samples was recorded.

Seawater samples were collected at the surface and drawn into 500 mL borosilicate glass bottles. Samples were preserved with 200 µL of saturated mercuric chloride and transported to CSIRO Marine Laboratories in Hobart, Australia, where they were analyzed for DIC and TA one month after collection. Salinity and temperature were measured during each sample using a YSI Castaway conductivity temperature depth (CTD) meter. Photosynthetically active radiation (PAR) was measured half-hourly during the sampling period at the main slack water sampling site with a Wet Labs EcoPAR sensor.

DIC and TA analyses were performed in accordance with the *Guide to Best Practices for Ocean CO_2_ Measurements* [30]. A Single-Operator Multi-parameter Metabolic Analyzer (SOMMA) was used for coulometric DIC determinations. TA was analyzed using the open cell method of acidimetric titration using a Metrohm Titrando. The precision of the analyses were determined through analysis of nine duplicate samples and resulted in a standard deviation of ±0.9 µmol kg^−1^ for TA and ±1.7 µmol kg^−1^ for DIC. Certified reference materials (CRMs) from Dr. A. Dickson at Scripps Institute of Oceanography were run at least twice daily to determine the accuracy of the analyses. Sample results were corrected based on the difference between the measured and certified values of the CRMs, where the average difference between measured and certified values for TA and DIC CRMs were −0.3 µmol kg^−1^ and −1.3 µmol kg^−1^ respectively.

### Calculations

G_net_ and P_net_ were calculated according to Equations 3 and 4 respectively, where ΔTA and ΔDIC are in mmol kg^−1^, Δt is in hours and depth is in meters. ρ (kg m^−3^) was calculated from salinity and temperature measurements [31]. F_CO2_ for the P_net_ calculation was calculated from Equation 5 [32].
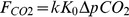
(5)where k is the gas transfer velocity, K_0_ is the solubility of CO_2_ in seawater and Δ*p*CO_2_ is the difference in partial pressure of CO_2_ (*p*CO_2_) between seawater and air. k was defined using the wind speed parameterization of Ho and colleagues [33]. Wind speed values, recorded every 10 min, were taken from the weather station at One Tree Reef [34]. Wind speed was measured at a height of 6 m and corrected to a height of 10 m for air-sea flux calculations [35]. Atmospheric CO_2_ concentration was assumed to be 394 µatm, which was the value from the Cape Grim monitoring station, assuming an atmospheric pressure of 1 atm. Seawater *p*CO_2_ was calculated from the measured salinity, temperature, DIC and TA, using CO2sys for MATLAB [36]. Calculations were made using the total hydrogen ion concentration scale, with the carbonic acid dissociation constants of Mehrbach *et al*. [37] as refit by Dickson and Millero [38] and the K_SO4_ for the bisulfate ion from Dickson [39].

Differences in P_net_ and G_net_ between the two methods were compared using paired t-tests, where observations were paired based on the hour that the sample was taken. In the case where there was an observation for only one of the methods in the hour, pairing could not occur and the value was excluded from the analysis. The assumption of normally distributed differences was confirmed through inspection of Q-Q plots of the differences. Statistical analyses were performed using SPSS version 21. P_net_ was split into separate “day” and “night” analyses to account for periods of net photosynthesis (6am–3pm) and net respiration (4pm–5am).

## Results

**Figure 2:**
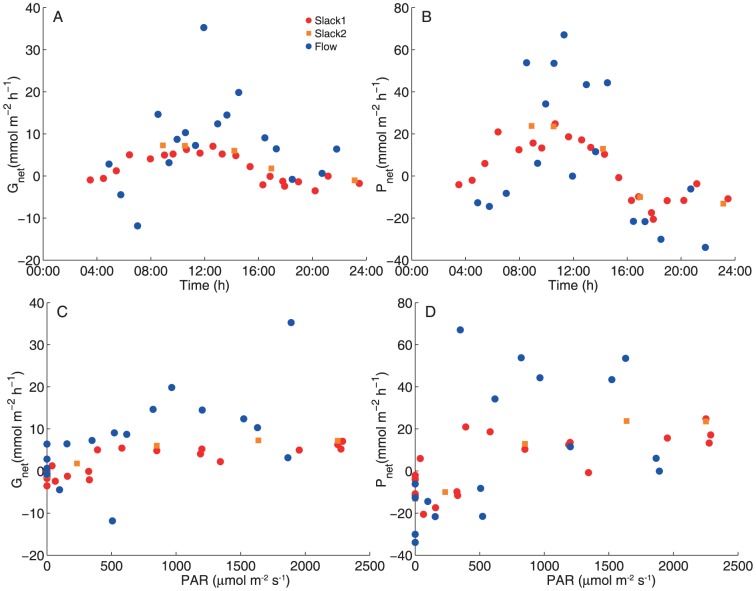
Variation of metabolic rates with time of day and PAR. (A) G_net_ and (B) P_net_ over time, and (C) G_net_ and (D) P_net_ versus PAR, for the slack water and flow respirometry methods, One Tree Reef flat, 8–22 November 2013. The main slack water sampling site is shown in red, the second slack water site in orange and the flow respirometry data shown in blue.

P_net_ values were higher during the day/light than night/dark regardless of the method used, as would be expected with net daytime photosynthesis and nighttime respiration (Fig. 2). G_net_ values were also higher during the day/light and lower at night/dark (Fig. 2), as is expected as a result of light-enhanced calcification [1]. Both G_net_ and P_net_ values calculated from the flow respirometry method showed larger variability than the slack water measurements (Fig. 2; Table 1). The G_net_ and P_net_ values at the second slack water site were similar to the values observed at the main slack water site (Fig. 2).

**Table 1:** Comparison of mean (±standard error) daytime and nighttime P_net_ and G_net_ values between the two methods.

	P_net_ (mmol m^−2^ h^−1^)		G_net_ (mmol m^−2^ h^−1^)	
	Day	Night	Day	Night
Slack water	6.2(3.8)	−7.3(1.8)	3.1(0.8)	−1.4(0.5)
Flow respirometry	19.1(8.7)	−16.6(6.6)	9.6(3.1)	1.8(1.3)

G_net_ from the flow respirometry measurements was significantly higher than from the slack water method (p<0.05; Table 1). Flow respirometry P_net_ was significantly lower at night than for slack water measurements (p<0.01). Average flow respirometry P_net_ values were higher during the day compared with the slack water method, however the differences were not statistically significant as a result of the large variability in the flow respirometry data (Table 1; Fig. 2).

## Discussion

### Comparison of methods

The flow respirometry and slack water methods have inherent differences that could be expected to lead to differences in metabolic measurements made using these techniques even at the same study site. The main differences are that the flow respirometry method integrates metabolic rates over a greater area along the transect, compared with the relatively small area of benthic cover influencing the point measurement for the slack water method, and the difference in water flow and the potential effects that this could have on metabolic rates. The similarity in P_net_ and G_net_ values between the two slack water sites indicates that the increased area covered by the flow respirometry method is not likely to be the main cause of the differences observed between the two methods in this study. However, this may not be the case in all locations, particularly where there are changes in the zonation of benthic community type over the length of the areas sampled, and thus spatial variability should be assessed.

A number of laboratory and chamber experiments, while not being perfect analogues for natural flow conditions, have shown higher metabolic rates under higher flow/stirring rates [21]–[23]. Therefore the increased flow rates associated with the flow respirometry method could be expected to increase rates of photosynthesis, respiration, calcification and dissolution. This could potentially result in higher rates of P_net_ and G_net_ during the day when photosynthesis and calcification are the dominant processes, and lower rates of P_net_ at night when respiration is dominant. The influence of increased flow on nighttime G_net_ would depend on whether calcification or dissolution is most affected. Daytime and nighttime G_net_ values from flow respirometry measurements were higher than from slack water measurements (Table 1), indicating a possible positive effect of increased water flow on calcification. P_net_ values were on average higher during the day and lower at night for the flow respirometry method (although daytime differences were not significant due to the large variability of the data; Table 1), as would be expected from higher flow rates resulting in higher rates of photosynthesis and respiration.

Part of the large variability in the flow respirometry data may also be due in part to the higher flow conditions. In a study on the effects of diffusion boundary layers and changes in pH on performance of a coralline macroalgae, Cornwall *et al*. [40] showed that organisms kept under high flow (thin diffusion boundary layer) had more variable growth and net calcification rates than those under low flow (thick diffusion boundary layer). Thick diffusion boundary layers may also be responsible for the lower metabolic rates in the slack water measurements, where diffusion boundary layers slow the uptake and release of compounds produced and consumed in metabolism (e.g. CO_2_, O_2_, nutrients). Although the results indicate that flow rate may be an important difference between the two methods, it is likely that some variation about the flow respirometry data is also due to uncertainties associated with this method (see below).

The flow respirometry method has the advantages of integrating a larger area and sampling during times of active water flow, but there are potential errors that could be introduced as a result of the flowing water conditions. In both Lagrangian and Eulerian flow respirometry, it is assumed that current flow is unidirectional, with some extent of lateral mixing of water acceptable where there are broad biological zones perpendicular to the transect [4]. Although a drifter was used to determine current flow and not dye, it is reasonable to assume that the flow of water in this study was unidirectional as a result of the strong tidal flow off the reef. On one sampling day where there were strong winds blowing in the opposite direction of the tidal flow, sampling did not occur due to potentially non-unidirectional flow. Furthermore, biological community zonation was perpendicular to the transect, further reducing the impact of any potential lateral mixing [4].

The flow respirometry method also assumes uniform flow throughout the water column, which may not be the case due to frictional effects of the reef matrix [41]. Tracking water flow with a drifter, as is commonly done in Lagrangian flow respirometry studies, may overestimate flow velocity of the water column if the upper layer of water moves faster than deeper water near the reef matrix [20]. In Eulerian flow respirometry studies, where current velocity is taken at the time of sampling, it is also possible to have erroneous values of water flow if the location of current measurement is not representative of the current profile of the transect.

Similarly, for the slack water method, assumptions regarding water movement can introduce error in metabolism calculations if not reasonably met. The slack water method assumes that advection and dispersion result in negligible changes to chemical properties and that there is no vertical gradient in changes to seawater chemistry. In order for the water column to be mixed such that there is no vertical gradient in seawater chemistry, there must be some level of advection. Shallow study sites, such as in this study (0.55 m), are likely to be reasonably vertically homogeneous. Although not measured here, previous studies by Shaw *et al*. [12] have shown vertical homogeneity at a similar reef flat study site. In this study we deployed a drifter at the slack water site and observed no visible movement during a 15 min deployment, indicating that the measured changes in seawater chemistry are likely influenced by processes occurring within a small area around the study site. This is further indicated by differences in measured TA and DIC between the two slack water sites, where samples collected within 10 min of each other at each site commonly had DIC and TA differences up to and exceeding 20 µmol kg^−1^ [27], indicating limited water mixing between the sites. It should be noted that this may not be the case during periods of stronger wind speeds and that levels of horizontal transport should be assessed at the time of study. At another site within the OTI reef, Silverman *et al*. [13] found no dye movement during some slack water occasions, but water flow of up to 1.8 cm s^−1^ on other occasions. Assumptions regarding horizontal and vertical mixing, whilst rarely assessed in metabolism studies, could be comprehensively evaluated through the deployment of an acoustic Doppler current profiler (ADCP).

### Sources of uncertainty

There are a number of terms used in the calculation of G_net_ and P_net_ (Equations 3 and 4), where error in each of these terms contributes to the overall error in P_net_ and G_net_. Here we examine possible sources of uncertainty both in this study and in the published literature. We examine ΔTA, ΔDIC, Δt, and d. The G_net_ term in the P_net_ equation is not explicitly examined as it is already examined in its components. F_CO2_ is also not examined as previous studies have found it to be small relative to the overall change in DIC [5], [42] and thus errors in F_CO2_ are likely to have minimal impact on the value of P_net_. In this study the average contribution of F_CO2_ to P_net_ was 0.17 mmol m^−2^ h^−1^, with maximum F_CO2_ of 1.6 mmol m^−2^ h^−1^. While any error in F_CO2_ likely contributes a very small amount to the overall P_net_ estimate, the larger F_CO2_ values do make a noticeable (albeit relatively small) contribution to P_net_ and should be included in the calculation. For our mean wind speed of 4.6 ms^−1^, F_CO2_ only exceeds 1 mmol m^−2^ h^−1^ when seawater pCO_2_ exceeds 900 µatm, with F_CO2_ being more significant at higher wind speeds. For comparative purposes it is noted that earlier studies [42], when F_CO2_ was less readily calculated, did not incorporate F_CO2_ in the calculation of P_net_.

The magnitude of ΔTA and ΔDIC are dependent on the community metabolism, water depth and the duration of time (Δt) between measurements. ΔTA and ΔDIC are expected to be greater where there is high community metabolism (e.g. through high levels of benthic cover of coral, algae, etc), shallow depth, where greater depth dilutes changes in DIC and TA that are mostly driven by benthic processes on coral reefs [43], and a longer duration between samples. If the change in DIC and TA concentrations are not substantially greater than the analytical uncertainty in the TA and DIC measurements, then the measurement uncertainty could constitute a significant proportion of ΔTA and ΔDIC. In this study the absolute values of ΔTA ranged from 0.5 to 33 µmol kg^−1^ (mean of 9 µmol kg^−1^) and 0 to 25 µmol kg^−1^ (mean of 11 µmol kg^−1^) for the slack water and flow respirometry methods respectively. Note that there will necessarily be some low ΔTA and ΔDIC values if measurements are made at times where P_net_ and G_net_≈0, so the focus is that the range of values and mean values are greater than the measurement error. This was the case here where the precision of TA measurements was ±0.9 µmol kg^−1^. Similarly, in this study ΔDIC ranged from 2 to 67 µmol kg^−1^ (mean of 15 µmol kg^−1^) and 3 to 59 µmol kg^−1^ (mean of 29 µmol kg^−1^) for slack water and flow respirometry respectively, with a measurement precision of ±1.7 µmol kg^−1^.

Although depth at a single point can be measured accurately, bottom depth can be variable over small spatial scales. Even for the slack water method, it was observed that bottom depth varied by up to 0.2 m within 1 m of the exact sampling position. It is estimated that there would be a depth uncertainty of ∼0.1 m for the study site used here (personal observation). Although not usually reported in studies of coral reef metabolism, Gattuso et al. [26] also reported a depth uncertainty of 0.1 m. For shallow environments, such as the study site here (slack water depth of 0.55 m), this uncertainty is quite large (18%). If the uncertainty is constant, such as for the slack water method where depth is always the same, then this is not a problem when comparing relative values of G_net_ or P_net_ under different environmental conditions or over different times of day, for example. However it is problematic when comparing metabolic rates between different locations. Depth for the flow respirometry method was taken as the average depth from the time of the two samples, which could have inaccuracies associated with the non-linear tidal change in depth. Although these inaccuracies are likely to be small given the small Δt, they could be a contributing factor to the larger variability in the flow respirometry data (Fig. 2).

Δt is able to be determined simply and accurately from sample times and thus should not be a source of error in the calculations. However, Δt is an important determinant of ΔTA and ΔDIC. If Δt is too short then ΔTA and ΔDIC may be sufficiently small that uncertainty in the measurements of TA and DIC become significant (see above). For flow respirometry, Δt values are dependent on the rate of current flow, which determines the time taken for the parcel of water to move from an upstream to downstream site, and thus may vary depending on the degree of wave or tide forcing and physical characteristics of the reef. As P_net_ and G_net_ are inversely proportional to Δt (Equations 3 and 4), so too is the error in P_net_ and G_net_. In this study the slack water measurements were taken with a Δt of 1 h (Table S1). Δt for flow respirometry varied from 0.2 to 0.8 h (Table S1), with a mean of 0.5 h. Thus for the average flow respirometry Δt of 0.5 h, the error in ΔTAdρ for G_net_ and ΔDICdρ for P_net_ (Equations 3 and 4) is doubled in comparison with slack water calculations with a Δt of 1 h. This may be one of the factors leading to the increased variability in the flow respirometry data compared with the slack water data (Fig. 2). As well as contributing to variable Δt, differences in flow rates between flow respirometry measurements could lead to differences in metabolic rates, where metabolism is dependent on flow rate. Therefore the inability to control flow rates during flow respirometry measurements may lead to higher variability in measured metabolic rates.

### Coral reef community metabolism studies

Table S1 summarizes values for ΔTA, TA precision, depth and Δt for studies that determined *in situ* G_net_ for coral reef environments using an alkalinity anomaly approach. Where P_net_ was calculated from measurements of DIC, the ΔDIC and precision of the DIC measurement were also recorded. Studies that did not report any of the above parameter values were excluded. Table S1 lists 12 studies that use the flow respirometry and 8 that use the slack water method. Other studies using different alkalinity anomaly techniques (e.g. control volume) were also included as it is still relevant to compare some parameters, such as ΔTA relative to TA precision.

**Figure 3:**
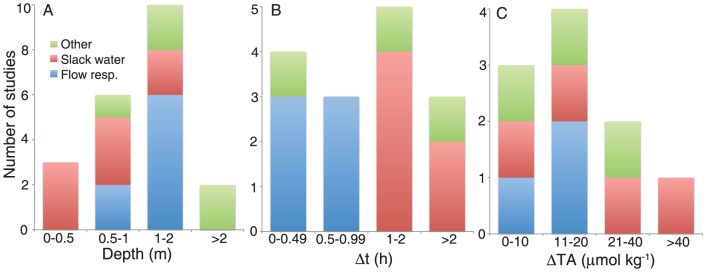
Summary of mean (A) depth (B) duration and (C) change in TA of metabolic studies using flow respirometry, slack water and other *in situ* methods of calculating net community calcification. Data is only shown for published studies in which mean parameters were provided or could be calculated. See Table S1 for the full dataset and further details.

Most studies that reported TA precision had values ≤2 µmol kg^−1^, with all reporting precision of ∼4 µmol kg^−1^ or less (Table S1). Most studies also reported ΔTA values much larger than the values of TA precision. ΔTA values are listed for six of the flow respirometry studies. The flow respirometry study of Smith [5] had average daytime changes in TA of ∼13 µmol kg^−1^, with a precision of 3.4 µmol kg^−1^, thus uncertainty in the measurement could be ∼25% of the ΔTA. The flow respirometry study of Albright et al. [15] reported low ΔTA values of 0–14 µmol kg^−1^ (mean of 4 µmol kg^−1^), however the precision of the TA measurements was not stated. The four slack water studies that have ΔTA values listed in Table S1 show relatively larger ΔTA (Fig. 3). Of the studies using other methods, most had reasonable changes in TA, relative to measurement precision, with the exception of the control volume study by Teneva et al. [14] that reported ΔTA values below the measurement precision 64% of the time. The nine studies that reported ΔDIC all had ΔDIC well above the measurement precisions listed (Table S1).

Of the 10 flow respirometry studies that reported depth, three had mean depths <1 m, six had a mean depth between 1 and 2 m, and one reported the depth varying from 1–4 m (Table S1). Six of the eight slack water studies were at depths of <1 m with the remaining two at a depth of 1.55 m (Fig. 3). Ten flow respirometry studies had a Δt value listed, all with mean Δt <1 h, apart from the study by Atkinson and Grigg [42] in the lagoon at French Frigate Shoals which was “up to 2 hours” with no mean value specified (Table S1). All of the six slack water studies reporting Δt were from 1–3 h, reflecting the amount of time that the study sites were isolated by the low tide and the desired temporal resolution. The generally shallower depths and longer Δt of the slack water studies (Fig. 3) is beneficial in contributing to higher ΔTA values. However, shallower depths make P_net_ and G_net_ estimates more susceptible to uncertainty in depth. Where measurements are made at a constant depth, such as in a slack water situation, depth uncertainty is not a problem for comparison between measurements at that site (e.g. examining changes in P_net_ or G_net_ with respect to *in situ* environmental parameters), as the relative error remains the same. However it is problematic for comparison between studies, where depth errors can lead to over- or under-estimation of G_net_ and P_net_ relative to other sites. For the flow respirometry method, where water depth can vary during the sampling period, measurement errors can also affect comparisons between G_net_ and P_net_ at that study site.

Several studies have related coral reef G_net_ to *in situ* changes in seawater calcium carbonate saturation state (Ω) [11], [12], [19], [44], where Ω is declining globally as a result of ocean acidification. Although these studies have found significant relationships between G_net_ and Ω, the relationships for any individual study site have not been applicable to other areas. In order to accurately predict how coral reef calcification may respond globally to threats such as ocean acidification, it is necessary to determine relationships between calcification and seawater carbonate chemistry that are applicable not only at the local scale. Differences between locations likely exist due to differences in community composition and physical processes. However, we show here that differences in methods and uncertainties within the methods could also influence relationships between community metabolism and environmental parameters. For example, a flow respirometry study in Kaneohe Bay [11] showed higher G_net_ values relative to Ω than for a slack water study on a GBR reef flat [12]. While this is undoubtedly due in part to differences in community composition and physical conditions between the sites and potential measurement uncertainties between the studies, part of the observed difference may also be a result of higher G_net_ values under the flowing conditions of the Kaneohe Bay study compared with the slack water conditions of the GBR study.

## Conclusions

Net community calcification, net daytime photosynthesis and nighttime respiration were higher using the flow respirometry method than the slack water method (although differences were not statistically different for daytime P_net_), likely due to differences in water flow between the two methods. There was larger variation in metabolic rates calculated using the flow respirometry method, which could be in part due to shorter Δt values, depth uncertainty between measurements, variable flow rates and/or thinner diffusion boundary layer thickness under high flow. A review of published coral reef community metabolic studies revealed that flow respirometry studies generally had lower ΔTA values, likely as a result of greater depth and lower Δt, and thus could be more susceptible to measurement uncertainty in TA constituting a notable proportion of the ΔTA value. Future community metabolic studies should aim to maximize the precision of TA and DIC measurements and ensure that study sites have sufficient ΔTA and/or ΔDIC over the specified time period (Δt) to maximize ΔTA and ΔDIC relative to measurement uncertainty, regardless of the method used. Shallow sites may maximize ΔTA and ΔDIC but precision of the depth measurement also has to be high to enable comparison between studies.

It is desirable to compare coral reef metabolism at different reefs, particularly in light of potential changes in G_net_ from global and local stressors, but as yet these relationships have not been applicable to other reefs [11], [12]. In order to compare studies that have used different methods, the methods must be directly comparable, with any methodological differences identified. We show here that differences may arise in the commonly used slack water and flow respirometry methods due to different flow conditions, and that each method is susceptible to different sources of uncertainty. The slack water method has the advantage of generally longer Δt than flow respirometry studies and a constant depth (leading to no relative depth error between measurements in the same study). The flow respirometry method is less well controlled in terms of depth, flow and Δt, but has the advantage of more realistic flow conditions. The large variability in flow respirometry data may complicate the formation of predictive relationships between G_net_, P_net_ and environmental parameters (e.g. Ω) and future studies using this technique should aim to identify and control factors that contribute to large variability in the data. Different physical processes between reefs (e.g. presence of unidirectional flow or times of slack water) necessitate that different methods will be used at different locations. Thus when comparing metabolic rates between locations it is important to consider that some difference may be due to the different methodologies. Studies applying the same methods at the same site over time will be useful in evaluating long-term changes in reef metabolism and will help limit these uncertainties.

## Supporting Information

Table S1 **Parameter values from coral reef metabolism studies.**
(DOCX)
