# Supplementary material for: Comparability of Slack Water and Lagrangian Flow Respirometry Methods for Community Metabolic Measurements
Source: PLoS One. 2014 Nov 26;9(11):e112161. doi: 10.1371/journal.pone.0112161 (PMC4245090; doi:10.1371/journal.pone.0112161)
Supplement: Table S1 — Parameter values from coral reef metabolism studies. (DOCX) [file pone.0112161.s001.docx]

| Reference | Site | Method | ΔTA  (μmol kg^-1^) | TA precision (μmol kg^-1^) | Depth (m) | Δt (h) | ΔDIC  (μmol kg^-1^) | DIC precision  (μmol kg^-1^) |
| --- | --- | --- | --- | --- | --- | --- | --- | --- |
| Gattuso et al. [[1996](#_ENREF_8)] | Moorea, French Polynesia | Lagrangian flow respirometry | 1-20 | ~2^1^ | 1.3 (mean) | 0.71 (mean) |  |  |
| Gattuso et al. [[1996](#_ENREF_8)] | Yong Reef, GBR | Lagrangian flow respirometry | 1-20 | ~2^1^ | 1.7 (mean) | 0.33 (mean) |  |  |
| Gattuso et al. [[1993](#_ENREF_9)] | Moorea, French Polynesia | Lagrangian flow respirometry |  | ~2^1^ | 1.4 (mean) | 0.28-0.85^2^ |  |  |
| Atkinson & Grigg [[1984](#_ENREF_2)] | Trig Is. reef flat, French Frigate Shoals, Hawaii | Lagrangian flow respirometry |  |  | 0-1 | 0.25-0.67 |  |  |
| Atkinson & Grigg [[1984](#_ENREF_2)] | Lagoon, French Frigate Shoals, Hawaii | Lagrangian flow respirometry |  |  | 1-4 | ≤2 |  |  |
| Albright et al. [[2013](#_ENREF_1)] | Davies reef flat, GBR | Lagrangian flow respirometry | 0-14  (mean = 4) |  | 1.8 (mean) | 0.28-1.87  (mean = 0.62) | 0-31  (mean = 10) |  |
| This study | One Tree reef flat, GBR | Lagrangian flow respirometry | 0-25  (mean = 11) | 0.9 | 0.8–1.6  (mean =1.1) | 0.2-0.8  (mean = 0.5) | 3-59  (mean = 29) | 1.7 |
| Barnes [[1983](#_ENREF_3)] | Davies Reef, GBR | Lagrangian flow respirometry |  |  | 0.83 (mean) | 0.42 (mean) |  |  |
| Barnes&Lazar[[1993](#_ENREF_4)] | Eilat, Red Sea | Lagrangian flow respirometry |  |  | 0.46-1.16  (mean = 0.75) | 0.13-0.25  (mean = 0.19) |  |  |
| Shamberger et al. [[2011](#_ENREF_15)] | Kaneohe Bay, Hawaii | Eulerian flow respirometry | 0-78 | ~2 | 2 (mean) |  | 0-94 | ~1 |
| Smith [[1973](#_ENREF_19)] | Eniwetok, Marshall Islands | Eulerian flow respirometry | ~13^3^ | ~3.4 |  | <1 |  |  |
| Kayanne etal. [[2005](#_ENREF_11)] | Palau barrier reef flat | Eulerian flow respirometry |  | 1.8 |  |  |  | 2.1 |
| Kinsey [[1972](#_ENREF_12)] | One Tree reef, GBR | slack water |  |  | 0.8 | ~3 |  |  |
| Ohde & van Woesik [[1999](#_ENREF_14)] | Rukan-sho, Okinawa | slack water | 2-267  (mean = 163) |  | 0.18 | ~3 | 66-284  (mean = 205) | 2-4^1^ |
| Hata et al. [[2002](#_ENREF_10)] | Ishigaki Is, Japan | slack water |  | 2 | 1.55 |  |  | 2 |
| Kayanne etal. [[2005](#_ENREF_11)] | Ishigaki Is, Japan | slack water |  |  | 1.55 | 1 |  |  |
| Shaw et al. [[2012](#_ENREF_16)] | Lady Elliot reef flat, GBR | slack water | 1-124  (mean = 32) | 1.8 | 0.4 | 1 | 3-138  (mean = 51) | 1.3 |
| Silverman et al. [[2012](#_ENREF_17)] | One Tree reef, GBR | slack water |  | 2 | 0.8 |  |  | 1.3 |
| This study | One Tree reef flat, GBR | slack water | 0.5-33  (mean = 9) | 0.9 | 0.55 | 1 | 2-67  (mean = 15) | 1.7 |
| McMahon et al. [[2013](#_ENREF_13)] | Heron reef flat, GBR | slack water^4^ | Mean ≈ 20 | <4^1^ | ~0.3-0.4^5^ | ~2 |  |  |
| Silverman et al. [[2007](#_ENREF_18)] | Nature Reserve Reef, Northern Red Sea | alkalinity-anomaly residence-time | 8-21 | 2 | 1.5-1.8 |  |  |  |
| Bates et al. [[2010](#_ENREF_5)] | Hog Reef, Bermuda | alkalinity-anomaly residence-time | 6.5-65 |  | 6 (mean) |  | 0-57 | 0.4 |
| Falter et al. [[2012](#_ENREF_6)]^6^ | Ningaloo Reef, Western Australia | Control volume | 0-38  (mean = 15) | 2-3 | 1.55 | 0.6 (mean) | 32 (mean) |  |
| Zhang et al. [[2012](#_ENREF_22)]^6^ | Coral Bay, Ningaloo Reef | Control volume | 6 (mean) | 1 | 1.29 | 0.27 (mean) |  |  |
| Teneva et al. [[2013](#_ENREF_20)] | Palau back reef | Control volume | Not given^7^ | 3.4 | ~8 |  |  | 2 |
| Gattuso et al. [[1997](#_ENREF_7)] | Moorea, French Polynesia | Enclosure |  |  | 0.45-0.78 | 1 |  |  |
| Yates & Halley [[2006](#_ENREF_21)] | Molokai reef flat, Hawaii | Enclosure | 1-79  (mean = 22) | 0.9 | NA | 4 | 4-122  (mean = 53) |  |

^1^ Estimated based on precision reported as a percentage

^2^ Estimated based on reported average transect length and range of current speeds

^3^ Calculated from the difference in average daytime upstream and downstream TA values

^4^ Note though that the reef was not fully enclosed and therefore water flow was occurring

^5^ Depth of 1.7 m specified in the publication as the mean depth of the Heron reef lagoon, however sampling site depth was 0.3-0.4m (I. Santos, pers. communication)

^6^ Information from this study was from the cited reference and personal communication with J. Falter

^7^ Although values were not given, the authors noted that ΔTA was below the instrumental precision 64% of the time

**References**

Albright, R., C. Langdon, and K. R. N. Anthony (2013), Dynamics of seawater carbonate chemistry, production, and calcification of a coral reef flat, central Great Barrier Reef, *Biogeosciences*, *10*(10), 6747-6758, doi:10.5194/bg-10-6747-2013.

Atkinson, M. J., and R. W. Grigg (1984), Model of a coral reef ecosystem, *Coral Reefs*, *3*(1), 13-22.

Barnes, D. (1983), Profiling coral reef productivity and calcification using pH and oxygen electrodes, *Journal of Experimental Marine Biology and Ecology*, *66*(2), 149-161.

Barnes, D., and B. Lazar (1993), Metabolic performance of a shallow reef patch near Eilat on the Red Sea, *Journal of experimental marine biology and ecology*, *174*(1), 1-13.

Bates, N. R., A. Amat, and A. J. Andersson (2010), Feedbacks and responses of coral calcification on the Bermuda reef system to seasonal changes in biological processes and ocean acidification, *Biogeosciences*, *7*(8), 2509-2530, doi:10.5194/bg-7-2509-2010.

Falter, J. L., R. J. Lowe, M. J. Atkinson, and P. Cuet (2012), Seasonal coupling and de‐coupling of net calcification rates from coral reef metabolism and carbonate chemistry at Ningaloo Reef, Western Australia, *Journal of Geophysical Research: Oceans (1978–2012)*, *117*(C5).

Gattuso, J. P., C. E. Payri, M. Pichon, B. Delesalle, and M. Frankignoulle (1997), Primary production, calcification, and air‐sea co2 fluxes of a macroalgal‐dominated coral reef community (Moorea, French Polynesia), *Journal of Phycology*, *33*(5), 729-738.

Gattuso, J. P., M. Pichon, B. Delesalle, C. Canon, and M. Frankignoulle (1996), Carbon fluxes in coral reefs .1. Lagrangian measurement of community metabolism and resulting air-sea CO2 disequilibrium, *Marine Ecology-Progress Series*, *145*(1-3), 109-121.

Gattuso, J. P., M. Pichon, B. Delesalle, and M. Frankignoulle (1993), Community metabolism and air-sea CO2 fluxes in a coral-reef ecosystem (Moorea, French-Polynesia), *Marine Ecology-Progress Series*, *96*(3), 259-267.

Hata, H., S. Kudo, H. Yamano, N. Kurano, and H. Kayanne (2002), Organic carbon flux in Shiraho coral reef (Ishigaki Island, Japan), *Marine ecology progress series*, *232*, 129-140.

Kayanne, H., H. Hata, S. Kudo, H. Yamano, A. Watanabe, Y. Ikeda, K. Nozaki, K. Kato, A. Negishi, and H. Saito (2005), Seasonal and bleaching‐induced changes in coral reef metabolism and CO2 flux, *Global biogeochemical cycles*, *19*(3).

Kinsey, D. W. (1972), Preliminary observations on community metabolism and primary productivity of the pseudo - atoll reef at One Tree Island, Great Barrier Reef, in *Proceedings of the First International Symposium on Corals and Coral Reefs*, edited by C. Mukudan and C. S. Gopinadha Pillai, pp. 13-32, Marine Biological Association of India, Mandapam Camp, India.

McMahon, A., I. R. Santos, T. Cyronak, and B. D. Eyre (2013), Hysteresis between coral reef calcification and the seawater aragonite saturation state, *Geophysical Research Letters*, *40*(17), 4675-4679.

Ohde, S., and R. van Woesik (1999), Carbon dioxide flux and metabolic processes of a coral reef, Okinawa, *Bulletin of Marine Science*, *65*(2), 559-576.

Shamberger, K. E. F., R. A. Feely, C. L. Sabine, M. J. Atkinson, E. H. DeCarlo, F. T. Mackenzie, P. S. Drupp, and D. A. Butterfield (2011), Calcification and organic production on a Hawaiian coral reef, *Marine Chemistry*, *127*(1-4), 64-75.

Shaw, E. C., B. I. McNeil, and B. Tilbrook (2012), Impacts of ocean acidification in naturally variable coral reef flat ecosystems, *Journal of Geophysical Research-Oceans*, *117*(C3), C03038, doi:10.1029/2011jc007655.

Silverman, J., D. Kline, L. Johnson, T. Rivlin, K. Schneider, J. Erez, B. Lazar, and K. Caldeira (2012), Carbon turnover rates in the One Tree Island reef: A 40‐year perspective, *Journal of Geophysical Research: Biogeosciences (2005–2012)*, *117*(G3).

Silverman, J., B. Lazar, and J. Erez (2007), Effect of aragonite saturation, temperature, and nutrients on the community calcification rate of a coral reef, *Journal of Geophysical Research-Oceans*, *112*(C5), doi:C05004

10.1029/2006jc003770.

Smith, S. (1973), Carbon dioxide dynamics: a record of organic carbon production, respiration, and calcification in the Eniwetok reef flat community, *Limnology and Oceanography*, 106-120.

Teneva, L., R. Dunbar, D. Mucciarone, J. Dunckley, and J. Koseff (2013), High-resolution carbon budgets on a Palau back-reef modulated by interactions between hydrodynamics and reef metabolism, *Limnol. Oceanogr*, *58*(5), 1851-1870.

Yates, K. K., and R. B. Halley (2006), CO32- concentration and pCO(2) thresholds for calcification and dissolution on the Molokai reef flat, Hawaii, *Biogeosciences*, *3*(3), 357-369.

Zhang, Z., J. Falter, R. Lowe, and G. Ivey (2012), The combined influence of hydrodynamic forcing and calcification on the spatial distribution of alkalinity in a coral reef system, *Journal of Geophysical Research: Oceans (1978–2012)*, *117*(C4).
